# Creativity, psychosis and human evolution: The exemplar case of *neuregulin 1* gene

**DOI:** 10.4103/0019-5545.71003

**Published:** 2010

**Authors:** Ganesan Venkatasubramanian, Sunil V. Kalmady

**Affiliations:** The Metabolic Clinic in Psychiatry, Department of Psychiatry, National Institute of Mental Health and Neurosciences, Bangalore–560029, India E-mail: venkat.nimhans@yahoo.com

Sir,

The link between creativity and psychosis has derived more support from a recent demonstration of a biologically relevant polymorphism of the promoter region of the *neuregulin 1* gene, which is linked with schizophrenia,[1] associated with creativity in people with high intellectual and academic performance.[2] In this letter, we summarize further comparative genomic analyses supporting positive selection of *neuregulin 1* gene that further adds to its significance in the context of creativity and psychosis from the perspectives of human evolution.

Comparative genomic analyses examine a fundamental measure of the relative importance of selection and genetic drift in causing amino-acid substitutions is the dN/dS ratio (dN is a measure of the degree to which two homologous coding Deoxyribonucleic acid (DNA) sequences differ at nonsynonymous sites, dS is a measure of the degree to which two homologous coding sequences differ with respect to silent nucleotide substitutions). Analyzing dN and dS are among the most direct ways to obtain evidence for positive selection on a protein-coding gene.[3] A comparative genomic research using sequences from 23 Eutherian species through phylogenetic analysis by maximum likelihood demonstrated compelling evidence for positive selection involving *neuregulin 1* gene.[4] This observation is in support of a previous comparative genomic analysis examining five species.[5] This adaptive change potentially dates to the evolution of modern humans i.e. five to seven million years ago.[4]

The intriguing persistence of schizophrenia (approximately 1% prevalence across all populations), despite adverse fecundity, argues for a balancing advantage conferred by this disorder that is shared by all human populations. Schizophrenia, a heterogeneous construct, is likely to be influenced by various balancing advantages–creativity being one of them.[6] While the recently reported link between neuregulin 1 and creativity[2] offers important evidence in support of this, other works (as summarized above) strengthen the evolutionary significance of *neuregulin 1* gene not only for schizophrenia, perhaps, but also for origins of *Homo sapiens*.

Dobzhansky’s statement: “Nothing in biology makes sense except in the light of evolution” emphasizes the need for evolutionarily-informed approaches to understand diseases and disorders. Contemporary medicine focuses predominantly on “proximal-etiology” whereas “distaletiology” based evolutionary approach has mostly been neglected.[7] ‘Theoretical’ research approaches (similar to Dr. Keri’s work)[2] based on evolutionary concept advocating multiple dimensions might facilitate identifying valid homogeneous subtypes that can advance our understanding of schizophrenia.[8]
